# Cytokines in hepatitis C-infected patients with or without opioid maintenance therapy

**DOI:** 10.1017/neu.2023.56

**Published:** 2024-01-04

**Authors:** Kristin Nygaard-Odeh, Hedda Soloy-Nilsen, Magnhild Gangsoy Kristiansen, Ole Lars Brekke, Tom Eirik Mollnes, Michael Berk, Jorgen G. Bramness, Terje Oiesvold

**Affiliations:** 1 Nordland Hospital Trust, Bodoe, Norway; 2 Institute of Clinical Medicine, UIT - The Arctic University of Norway, Tromsoe, Norway; 3 Research Laboratory, Nordland Hospital Trust, Bodoe, Norway; 4 Department of Immunology, Oslo University Hospital and University of Oslo, Oslo, Norway; 5 Deakin University, IMPACT – the Institute for Mental and Physical Health and Clinical Translation, School of Medicine, Barwon Health, Geelong, Australia; 6 The National Centre of Excellence in Youth Mental Health, The National Centre of Excellence in Youth Mental Health, Centre for Youth Mental Health, The University of Melbourne, Melbourne, Australia; 7 Norwegian National Advisory Unit on Concurrent Substance Abuse and Mental Health Disorders, Innlandet Hospital Trust, Brumunddal, Norway; 8 Department of Alcohol, Tobacco and Drugs, Norwegian Institute of Public Health, Oslo, Norway

**Keywords:** Cytokines, hepatitis, inflammation, opioid maintenance treatment, chemokines

## Abstract

**Objective:**

Both chronic hepatitis C virus (HCV) infection and opioids cause altered blood levels of cytokines. Previous studies have investigated levels of selected groups of cytokines in patients on opioid maintenance treatment. Little is known about the levels of multiple cytokines in patients with chronic HCV infection on opioid maintenance treatment. Our aim was to investigate the cytokine profile in patients with active HCV infection with and without opioid maintenance treatment.

**Methods:**

We conducted a cross-sectional study in an out-patients population included upon referral for antiviral hepatitis C infection treatment. The level of 27 cytokines was measured in serum using multiplex technology. Patients were interviewed using a modified version of the European addiction severity index. Data pertaining to weight, height, current medication, smoking habits, allergies, previous medical history and ongoing withdrawal symptoms were collected. Non-parametric testing was used to investigate differences in levels of cytokines between the two groups. A 3-model hierarchical regression analysis was used to analyse associations between cytokines and confounding variables.

**Results:**

Out of 120 included patients, 53 were on opioid maintenance treatment. Median duration of opioid treatment was 68.4 months. There were no demographical differences between the two groups other than age. IL-1β was lower and eotaxin-1 higher in the group on opioid maintenance treatment than in the non-opioid group. No other inter-group differences in the remaining cytokine levels were found.

**Conclusion:**

In HCV infection patients, the impact of chronic opioid administration on peripheral circulating cytokine level is minimal.

## Significant outcomes

- No demographic differences other than age were found between the OMT and non-OMT group, both groups with chronic HCV infection.
- Of the 27 cytokines, only IL-1β (lower) and eotaxin-1 (higher) displayed differences in levels in the OMT patients compared to the non-OMT patients.

## Limitations

- Too many data were missing for factors impacting cytokine levels (intake of anti-inflammatory and/or psychotropic drugs, smoking) which thus could not be corrected for.
- The study lacks a healthy, non-HCV-infected control group.

## Introduction

Opioids are essential for the treatment of acute pain, chronic cancer pain and palliative treatment. While effective in these situations, opioids also have adverse and unintended effects such as abuse and dependence (Bailey and Connor, 2005). It has been calculated that more than 40.5 million people globally are opioid-dependent (Degenhardt et al., 2019), causing opioid dependence to account for 9.5 million disability-adjusted life years (Degenhardt et al., 2014). Illegal opioids and especially heroin are commonly used by people who inject drugs (Degenhardt et al., 2019; Gjerde et al., 2021). In 2013, 517 000 individuals aged 12 or older were dependent on or abused heroin in the U.S (Lipari and Hughes, 2013), and in Norway, heroin is the most commonly injected illicit drug (Gjerde et al., 2021). The drugs of choice used in treating opioid dependence are the long-acting opioids methadone and buprenorphine. At the end of 2021, more than 8000 patients were on opioid maintenance therapy (OMT) in Norway (Bech, 2022). Approximately one-third received methadone and two-thirds some form of buprenorphine-formulation.

Opioids exert their effects through the opioid receptors present in many brain regions, the peripheral nervous system, the gastrointestinal tract and the immune system (Brejchova et al., 2020). Early reports demonstrated an immunosuppressive effects of morphine (Yeager et al., 1995), and further studies on the immunomodulatory effects of other analgesic opioids have also mostly been performed in acute, controlled postsurgical settings (Sacerdote et al., 2000; Yardeni et al., 2008, Cui et al., 2017). Studies on chronic illicit opioid use, mostly heroin, have found altered cytokine levels compared to healthy adults (Sacerdote et al., 2008). This alteration does not appear to be affected by transitioning from illicit use to controlled heroin-assisted treatment (Hansen et al., 2021). Reports on the immune effect of transitioning from heroin use to OMT show conflicting results (Sacerdote et al., 2008; Wang et al., 2018; Lu et al., 2019). However, the duration of treatment at the time of cytokine measurements differ, and data suggest that duration of treatment and cytokine levels have a positive correlation (Chan et al., 2015).

Hepatitis C Virus (HCV) infection is globally affecting 2.8% of the population (Petruzziello et al., 2016), and prevalence among patients on OMT is 43% (Schulte et al., 2020). The Norwegian government has a strategy of reducing HCV by 90%; hence, all infected persons are eligible to receive antiviral therapy upon referral to infectious disease outpatient clinics. HCV affects levels of circulating cytokines (Capone et al., 2014). Studies comparing HCV patients with healthy controls found elevated levels of IL-1 α, IL-1β, IL-6, IL-8 (Costantini et al., 2010) and IL-1 α, IL-2R, IL-12, IL-18 (Costantini et al., 2012).

Because of the high the prevalence of HCV infection among people on OMT, it is of interest to study a wider panel of cytokines than previously investigated and to identify the inflammatory profile in these patients. This will give us the opportunity to see whether chronic administration of opioids influences the immune system in a way that is visible even in the immune-activated HCV patients. Thus, the aim of this study is to investigate the inflammatory response using a multiplex platform of cytokines, including interleukins, chemokines, interferons and growth factors in an OMT-outpatient vs. non-OMT population, both of which have active HCV infection.

## Materials and methods

### Design, recruitment and participants

In this cross-sectional study, patients referred for anti-HCV treatment were recruited from the Department of Infectious Diseases at the Nordland Hospital Trust, Bodoe, Norway, in the period of April 2013-December 2019. On the day of their appointment, the patients were approached by the study nurse who briefly informed them about the study and asked if willing to participate. Those willing subsequently met with the main investigator (first author) and were given verbal and written detailed information of the study, and written consent was obtained. The study was approved by the regional ethics committee (notification 2015/1808/REK Nord). Exclusion criteria were patients not understanding the Norwegian language, obvious cognitive deficits, negative HCV-RNA, failure to obtain written consent and failure to obtain blood samples. Out of the 155 screened patients, 35 were excluded for not meeting the requirements. Fifty-three patients (cases) were on OMT, and 67 patients (controls) were not.

### Data collection

Upon recruitment, the patients were interviewed by the main investigator using the protocol’s modified adaptation of a multidimensional assessment instrument for drug and alcohol dependence, the European Addiction Severity Index (Europ-ASI) questionnaire. The Europ-ASI is a multidimensional instrument (Ahmad-Nielsen et al., 2019). In our adapted version, we extrapolated the parts general information, physical health, economy, education and employment status and alcohol and other drugs use. Interviewer’s severity assessment and reliance assessment were omitted. For the cases, information pertaining to the type of maintenance drugs and dosage was collected. Gender and age were obtained from the person identification data, and their weight was measured by the study nurse at the day of inclusion. Patients were interviewed concerning other current medication, smoking habits, height, allergies, previous medical history and ongoing withdrawal symptoms. Body mass index (BMI) was calculated from the formula BMI = kg/m^2^.

### Blood sampling and analyses

Blood samples were drawn by the study nurse on the day of inclusion. All blood withdrawals were performed between 1100 am until 0250 pm. Biochemical measures were performed at the Department of Laboratory Medicine, Nordland Hospital Trust. For measurement of serum cytokines, blood was withdrawn in Vacuette serum tubes, left for 30 minutes before centrifugation 10 minutes at 2300x g (3500 r.p.m.). Serum (2x 1 mL) was stored in Matrix tubes on ice up to 2 hours before freezing at −80^o^ C.

Cytokine analyses were performed by multiplex technology with a predefined kit Bio-Plex Human Cytokine 27-Plex Panel (Bio-Rad Laboratories Inc., Hercules, CA) according to the instructions of the manufacturer. The assay detected the following interleukins, chemokines and growth factors: tumour necrosis factor (TNF), interferon (IFN)-gamma, IL-1β, IL-1 receptor antagonist (IL-1ra), IL-2, IL-4, IL-5, IL-6, IL-7, IL-8 (C-X-C motif chemokine ligand 8; CXCL8), IL-9, IL-10, IL-12, IL-13, IL-15, IL-17, monocyte chemotactic protein (MCP-1) or CCL2, interferon-inducible protein (IP-10) or (C-X-CL chemokine 10; CXCL10), eotaxin-1 (C-C motif chemokine ligand 11; CCL11), macrophage inflammatory protein-1 *α* (MIP-1 *α* or CCL3), macrophage inflammatory protein-1-*β* (MIP-1*β* or CCL4), regulated upon activation T cell expressed and secreted (RANTES), granulocyte macrophage-colony stimulating factor (GM-CSF), vascular endothelial growth factor (VEGF), basic fibroblast growth factor (bFGF), granulocyte-colony stimulating factor (G-CSF) and platelet-derived growth factor-BB (PDGF-BB).

## Statistical analyses

Comparisons of the variables between the cases and controls were performed using Mann–Whitney *U*-test for continuous vs. binary variables and non-normal distributed variables, and chi-square tests for categorical variables. *P*-values < 0.05 were deemed statistically significant. Tests for cytokine distribution were performed by Q-Q plots and found to be normal with lg10 transformation. Extreme outliers defined as lying outside the third quartile + 3* interquartile range were identified by boxplots. Their impact was tested using independent-samples *t*-test before and after their removal. Cytokine values below lower limit of detection (LLOD) were assigned a value randomly drawn by Excel between LLOD and zero. The cytokines with more than 50% randomly drawn numbers were excluded from further analyses, excluding β-FGF, IL-7, IL-10 and G-CSF. Hierarchical regression analysis was performed to adjust for confounding factors in a 3-model way. Testing with variance inflation factor below 10, showed no problems with collinearity. Statistical analyses were performed using IBM SPSS Statistics viewer version 28.0.1.0.

## Results

Out of the 120 patients included with active hepatitis C infection, 53 were on OMT (Table 1). Median duration of treatment was 68.4 (IQR 14.4-120) months. The median age of cases (42 years) was lower than that of controls (47 years) (*p* = 0.043). No other significant difference in the variables depicted in Table 1 was found, including other demographic variables, virus load and drug use during the last 30 days.

**Table 1.** Difference between OMT^1^ and non-OMT patients in demographic characteristics, virus load and substance use last 30 days

		OMT	Non-OMT	*p*-value
*Demographics*		*N = 53 (44%)*	*N* = 67 (56%)	*p*-value
Female gender	N (%)	13 (25)	26 (39)	0.097
Age (years)	Median (IQR)	42 (34–51)	47 (37–56)	**0.043**
On psychotropic drugs	N (%)	19 (35)	15 (22)	0.236
BMI	Median (IQR)	26 (23–30)	25 (22–28)	0.421
Smoking	N (%)	32 (61)	39 (58)	0.140
Norwegian nationality	N (%)	47 (89)	60 (90)	0.462
**Virus load**				
HCV-RNA	Median (IQR)	633000 (276000–3109500)	1255000 (435000–3297000)	0.154
**Substance use last 30 days**				
Alcohol	N (%)	20 (43)	35 (53)	0.272
Cannabis	N (%)	21 (44)	20 (33)	0.268
Amphetamine	N (%)	4 (9)	9 (17)	0.222
Benzodiazepine	N (%)	17 (39)	11 (26)	0.218
Heroin	N (%)	1 (2)	1 (3)	0.882
Other opioides	N (%)	1 (2)	3 (8)	0.229

1 Abbreviations: BMI = body mass index; HCV = hepatitis C virus; OMT = opioid maintenance therapy.

Comparing the opioid-substituted cases with controls, two cytokines showed significantly different median levels: IL-1*β* was lower (*p* = 0.017) and eotaxin-1 higher (*p* = 0.015) among those using opioids (Table 2). Outliers were not found to have an impact on the median value of the cytokines.

We ran a 3-model approach in the hierarchical regression analysis: model A for the unadjusted association between OMT and the two cytokines, model B adjusting for age, gender and BMI and model C adjusting for age, gender, BMI and the opposing cytokine (Table 3). For IL-1β, there was a significant association in the unadjusted model A between cases and the cytokine (*β* = 0.2, *p* = 0.048). The association remained significant after adjustment in both model B (*β* = 0.19, *p* = 0.038) and model C (*β* = 0.25, *p* = 0.005). There was also a significant association between duration of OMT and IL-1*β* (*p* = 0.049).

**Table 2.** Cytokine levels in non-OMT^1^ vs. OMT patients

	Non-OMT	OMT	
Cytokine	Median (IQR)	Median (IQR)	*p*-value^2^
TNF	48 (40–62)	50 (41–67)	0.411
IFN-γ	3.3 (0.9–8.3)	3.34 (1.40–8.33)	0.693
IL-1β	0.46 (0.31–0.85)	0.59 (0.35–1.13)	**0.017**
IL-1ra	148 (121–196)	148 (111–227)	0.983
IL-2	1.9 (1.1–2.6)	1.7 (1.2–2.6)	0.859
IL-4	1.8 (1.0–2.6)	1.7 (1.0–2.5)	0.745
IL-5	4.7 (2.3–6.3)	4.1 (2.0–7.1)	0.401
IL-6	1.0 (1.0–2.0)	1.0 (1.0–2.0)	0.139
IL-8	7.0 (5.0–12)	9.0 (6.0–12)	0.152
IL-9	6.0 (4.0–11)	6.0 (5.0–8.0)	0.825
IL-12	0.8 (0.2–2.0)	0.7 (0.2–0.9)	0.062
IL-13	1.5 (0.9–2.1)	1.5 (0.9–2.3)	0.520
IL-15	11 (5.7–31)	15 (3.5–24)	0.954
IL-17	5.6 (3.8–8.2)	4.9 (3.6–6.6)	0.253
MCP-1	36 (20–61)	33 (20–58)	0.899
IP-10	251 (146–412)	208 (111–415)	0.521
Eotaxin-1	100 (76–145)	80 (48–111)	**0.015**
MIP-1α	1.4 (1.1–2.2)	1.6 (1.2–2.1)	0.593
MIP-1β	127 (107–141)	133 (123–143)	0.161
RANTES	25,148 (13,104–29392)	26,399 (14,624–35774)	0.257
GM-CSF	0.84 (0.61–1.3)	0.98 (0.61–1.2)	0.775
VEGF	43 (33–54)	43 (38–53)	0.977

1 Abbreviations: OMT = opioid maintenance therapy (for cytokines: see main manuscript).

2 Independent-samples Mann–Whitney *U*-test.

**Table 3.** Hierarchical regression analysis of the association between being on OMT^1^ and serum levels of cytokines IL-1β and eotaxin-1 with reference to non-OMT patients

	IL-1*β*			Eotaxin-1		
	Coef.^2^	95% CI	*p*-value	Coef.^2^	95% CI	*p*-value
Model A: Unadjusted analysis	0.17	−0.00, 0.33	**0.048**	−0.09	−0.19, 0.02	0.112
Model B: Adjusted for age, gender, BMI	0.19	0.01, 0.33	**0.038**	−0.11	−0.21, 0.00	0.058
Model C: Adjusted for age, gender, BMI, opposite cytokine^3^	0.25	0.08, 0.42	**0.005**	−0.15	−0.25, −0.04	**0.007**

1 Abbreviations: OMT = opioid maintenance therapy. CI = confidence interval. BMI = body mass index.

2 Values are the log10-transformed.

3 IL-1β and exotaxin-1, respectively.

For eotaxin-1, model A (*β* = −0.86, *p* = 0.112) and model B (*β* = −0.11, *p* = 0.058) caused a loss of significance in the hierarchical regression analysis (Table 3). Model C showed association after adjusting for all the variables (*β* = −0.15, *p* = 0.007).

Further analysis was performed on the association between the cytokines and dosage of maintenance medication converted to morphine-equivalents, but none was found (IL-1*β*: *p* = 0.442, eotaxin-1: *p* = 0.268).

## Discussion

In this study of patients with chronic hepatitis C infection and OMT, we investigated the levels of a multiplex of 27 cytokines. We found that the level of cytokine IL-1β was lower and eotaxin-1 was higher in patients on OMT compared to a non-OMT group. Median duration of maintenance treatment was 68.4 months. The findings remained statistically significant after correcting for age, gender, BMI and the other significant cytokine.

Several clinical studies in adults have investigated levels of cytokines in patients on OMT (Neri et al., 2005; Chan et al., 2015; Wang et al., 2018; Kuo et al., 2018; Schroeder et al., 2018; Salarian et al., 2018; Lu et al., 2019). Studies investigating levels of IL-1β show different results compared to our results. In patients with methadone treatment, no significant change in level was found at 12 weeks compared to that at weeks 1, 4 and 8 (Lu et al., 2019). Others found higher levels at 10-12 weeks (Neri et al., 2002) and at 24 months (Chan et al., 2015). The level was correlated with duration of treatment (Chan et al., 2015). The level of IL-1β was also higher after 12 months of either buprenorphine or methadone maintenance treatment, with no significant difference between the two drugs (Neri et al., 2005).

IL-1β is a pro-inflammatory cytokine produced by cells of myeloid origin, and whose level is tightly regulated (Van Den Eeckhout et al., 2021). Increased levels have been implicated in several illnesses. Our finding with lower levels after long-term opioid treatment may thus have clinical relevance in conditions such as lymphomas (Sarani et al., 2021), breast cancer (Tulotta et al., 2019) and squamous cell carcinoma of the lungs (Suzuki et al., 2021).

Eotaxin-1 is a chemotactic agent, the elevated levels of which lead to eosinophilic chemoattraction (Zajkowska and Mroczko, 2021). Higher levels of eotaxin-1 have consequently been found in eosinophilic conditions such as asthma (Paplińska et al., 2012) and allergic rhinitis (Paplińska et al., 2012). Moreover, higher levels of eotaxin-1 have been associated with both neuroinflammatory (Huang et al., 2020) and neurodegenerative (Morgan et al., 2019) conditions. In addition, it has been associated with ageing (Villeda et al., 2011) and might be a marker of accelerated ageing (Panizzutti et al., 2015). The number of clinical studies performed in adult patients on OMT investigating levels of eotaxin-1 is limited. Consistent with our findings, eotaxin-1 level was higher in methadone-treated patients compared to the healthy control group, but in an age-related manner (Kuo et al., 2018).

### The implication of elevated levels in patients on OMT needs further investigation

None of the other cytokines in the multiplex displayed significant inter-group differences in their levels. Other studies have investigated IL-4 (Sacerdote et al., 2008), IL-6 (Wang et al., 2018; Lu et al., 2019; Hansen et al., 2021), TNF (Sacerdote et al., 2008; Salarian et al., 2018; Wang et al., 2018) and IFN-γ (Sacerdote et al., 2008; Salarian et al., 2018; Hansen et al., 2021). They report on the restorative immunological effects brought about by transitioning from illicit opioid use to OMT and hence differ from our study. In addition, cytokine levels were not compared to those of a control group, but to baseline levels (illicit opioid use). Other transitional studies in which levels of cytokines in OMT patients are compared to that of controls, showed higher levels of IL-6 and IL-8 (Chan et al., 2015), and TNF and IL-2-β (Neri et al., 2002). Mean duration of maintenance treatment was 23.6 months (Chan et al., 2015) and 3 weeks (Neri et al., 2002).

In addition to duration and type of treatment, there are other demographical and methodological differences between our study and the above-mentioned. All of these studies were performed on a select small group of cytokines. We have not found any other studies investigating such a wide array of cytokines in a population of OMT. Whereas our cytokine analysis was performed on serum, other studies were performed on plasma (Neri et al., 2005; Chan et al., 2015; Kuo et al., 2018; Wang et al., 2018; Schroeder et al., 2018; Salarian et al., 2018; Lu et al., 2019) or by specific solid-phase enzyme-linked assays (Neri et al., 2002). Thus, different methods of cytokine detection may explain contradictory results. There is a high prevalence of hepatitis C infection in patients on OMT (Schulte et al., 2020), and cytokine levels are known to be altered in patients with hepatitis C (Lapiński, 2001; Shrivastava et al., 2013; Rios et al., 2021). Infection status was unknown in several of the other studies (Neri et al., 2005; Chan et al., 2015; Kuo et al., 2018; Lu et al., 2019) and hence not corrected for.

### Limitations

The study has several limitations: Cytokine levels are known to be influenced by anti-inflammatory drugs(Koj, 1998), psychotropic drugs (Hernández et al., 2008; Stapel et al., 2018; Chen et al., 2018) and smoking (D’Esposito et al., 2022). Too many data were missing for these variables and could thus not be corrected for. In order to shed further light on the contribution that different factors have on cytokine levels, a healthy control group would have strengthened the results. As described previously, time duration of the OMT varies between the different reports. If the duration of therapy is of significance (Chan et al., 2015), a longitudinal study instead of cross-sectional would be more feasible. Blood samples were collected within a timeframe spanning over 3 hours, and diurnal variations in cytokine levels could have an impact (Liu et al., 2006; Nguyen et al., 2013).

### Strengths

Both groups in our study had chronic hepatitis C infection. The study design offers therefore an inherent statistical correction for the impact chronic hepatitis C has on cytokine levels. Given the prevalence of HCV in OMT patients, this provides for a valid, representative patient population.

We performed an analysis on a multiplex of 27 cytokines. As cytokines do not act in closed, isolated systems but rather on a backdrop of many influencing factors (Litteljohn and Hayley, 2012), analysing many cytokines rather than a select few facilitates a representative construct.

The protocol had a stringent design in which all patients were interviewed by the same person (first author), eliminating inter-rater variability. Blood samples were withdrawn by the same two, experienced nurses, and immediately transported to the laboratory facilities after withdrawal. This eliminates the impact delayed processing of whole blood into serum has on cytokine levels (Lee et al., 2016).

## Conclusion

In this multiplex assay of 27 cytokines in patients with chronic hepatitis C infection, levels of IL-1β were lower and eotaxin-1 higher in the patients on OMT compared to the non-OMT patients. None of the other cytokines displayed significant inter-group variances. To investigate the potential of immunological effects in long-lasting opioid treatment, longitudinal studies are warranted.
